# Geographic disparities in access to cancer clinical trials in India

**DOI:** 10.3332/ecancer.2021.1161

**Published:** 2021-01-05

**Authors:** Santam Chakraborty, Indranil Mallick, Hung N Luu, Tapesh Bhattacharyya, Moses Arunsingh, Rimpa Basu Achari, Sanjoy Chatterjee

**Affiliations:** 1 Department of Radiation Oncology, Tata Medical Center, Kolkata 700156, India; 2 Division of Cancer Control and Population Sciences, UPMC Hillman Cancer Center, University of Pittsburgh, Pittsburgh, PA 15232, USA; 3 Department of Epidemiology, Graduate School of Public Health, University of Pittsburgh, Pittsburgh, PA 15232, USA

**Keywords:** geography, neoplasms, disparities, access, clinical trials

## Abstract

**Introduction:**

The current study was aimed at quantifying the disparity in geographic access to cancer clinical trials in India.

**Methods:**

We collated data of cancer clinical trials from the Clinical Trial Registry of India and data on state-wise cancer incidence from the Global Burden of Disease Study. The total sample size for each clinical trial was divided by the trial duration to get the sample size per year. This was then divided by the number of states in which accrual was planned to get the sample size per year per state (SSY).

For interventional trials investigating a therapy, the SSY was divided by the number of incident cancers in the state to get the SSY per 1,000 incident cancer cases. The SSY data was then mapped to visualise the geographical disparity.

**Results:**

We identified 181 ongoing studies, of which 132 were interventional studies. There was a substantial inter-state disparity—with a median SSY of 1.55 per 1,000 incident cancer cases (range 0.00–296.81 per 1,000 incident cases) for therapeutic interventional studies. Disparities were starker when cancer site-wise SSY was considered. Even in the state with the highest SSY, only 29.7% of the newly diagnosed cancer cases have an available slot in a therapeutic cancer clinical trial. Disparities in access were also apparent between academic (range: 0.21–226.60) and industry-sponsored trials (range: 0.17–70.21).

**Conclusion:**

There are significant geographic disparities in access to cancer clinical trials in India. Future investigations should evaluate the reasons and mitigation approaches for such disparities.

## Introduction

Advances in cancer diagnosis, treatment and outcomes stem from well-designed clinical trials. Intuitively, patient participation in clinical trials is vital, but several studies show that less than 5% of adult cancer patients participate in a clinical trial [1–4]. Even where industry-sponsored research is concerned, a recent meta-analysis showed a marginally higher participation rate of 8% [5]. Key patient-reported barriers which limit participation are a dislike of randomisation, protocol complexity, presence of placebo arm, potential side effects, the inappropriateness of clinical trials in serious diseases and the effect on the relationship with healthcare providers [6]. Additionally, structural and clinical barriers exist, and non-availability of clinical trials was identified as one of the most critical factors affecting recruitment in clinical trials by Unger *et al* [5]. Nearly 55% of the patients did not have a trial available for them for participation, and an additional 21% were ineligible. Thus, geographical access to a clinical trial may impact recruitment into clinical trials. Globally, the disparity in access to cancer clinical trials is magnified in lower-middle-income countries (LMIC) [7]. As opposed to nearly 4,700 cancer clinical trials in breast, lung and cervical cancers in high-income countries, there were only 1,951 clinical trials available in LMIC nations [7].

India is a populous nation with an increasing burden of cancer. Recent data from population-based cancer registries (PBCR) indicate that the age-adjusted annual incidence of cancer is 94.1 per 100,000 males and 103.6 per 100,000 females [8]. In absolute numbers, this translates into 1,392,719 incident cases [8]. Additionally, there is significant geographic variability in the incidence and types of cancers. India has the third-highest number of incident cases worldwide (behind China and the USA) and is the second in terms of the number of deaths (665,650 in 2019) [9].

Despite the patient burden, few cancer clinical trials are available in India. A recent audit of clinical trials registered in the Clinical Trial Registry of India (CTRI, www.ctri.nic.in) showed that only 350 interventional trials were registered during 2007–2017 period [10]. Given the large size and wide disparity in health care services across India, there may be wide geographic variations in access to clinical trials also. The objective of the current study was to quantify the geographic disparities in access to clinical trials in India in terms of positions or slots in clinical trials available for newly diagnosed cancer patients.****

## Methods

### Clinical trial data

Data regarding registered clinical trials in cancer were retrieved from the CTRI website between 15 July 2020 and 17 July 2020. Both the simple search and advanced search facility available on the Website were used. Keywords searched for were ‘cancer’, ‘neoplasm’, ‘tumour’, ‘tumour’, ‘carcinoma’ and ‘sarcomas’. No restriction or filtering was used during the search. Search results were available on a hypertext mark-up language (HTML) page containing tabular summaries of the trials. Links to individual pages describing the trial were then obtained, and web data were extracted from the respective HTML pages. Extracted data on the clinical trial included information on the following parameters, amongst others:

1. Title and investigator names
2. Type of trial—Interventional (I), Observational (O), Bioavailability and Bioequivalence (BA/BE) and post-marketing surveillance (PMS) studies
3. Recruitment status
4. Health condition studied
5. Intervention(s) and comparator(s) as applicable
6. Sponsor type
7. Trial design and phase
8. The planned sample size for India
9. Date enrolment started and total trial duration
10. Multinational/Indian study

We then filtered these trials by their recruitment status, retaining only trials which were marked as open to recruitment. Furthermore, we also computed the planned date of closure of the study based on the total trial duration and the date of first enrolment. Trials that were indicated as open but had a projected date of closure earlier than 1 January 2020 were also excluded. Finally, trials which were unrelated to cancer were removed. Studies primarily sponsored by pharmaceutical companies were considered industry-sponsored, and rest were considered academic.

For each of these trials, we then manually abstracted the following information related to the trial:

1. Whether the study was related to cancer:
2. The cancer site(s)—trials recruiting patients across sites were categorised as multiple sites.
3. Stage of disease: Categorised into metastatic, non-metastatic and unstaged (haematological malignancies, sarcoma and brain tumours).
4. Preventive trial: Coded as Yes or No based on whether the trial was related to primary or secondary prevention of cancer in a healthy population.
5. Treatment intent: Was coded as curative or palliative or both based on the setting in which patients were treated.
6. Treatment setting: Was coded as Definitive, Adjuvant/Neoadjuvant, Palliative and Mixed based on the setting in which the study treatment was delivered.
7. Treatment type: Type of treatment modality, viz. Surgery/Radiotherapy/Systemic therapy/Supportive Care and Palliative Care. Patients who received chemotherapy, endocrine therapy or targeted therapy were categorised to be receiving systemic therapy.
8. Complementary or alternative medicines (CAM) therapy—If the trial was related to CAM, this was marked as yes.

The names of the Indian states where the trial was open were extracted from the site addresses where available. In case the states were not available, then reverse geocoding of the addresses was done to obtain information on the state. For each trial, we calculated the number of states in which the trial was open. The total sample size available in India was divided by the total trial duration in years to obtain the sample size available for recruitment per year (SY). The SY was divided equally amongst the states in which the trial was to be conducted to get the sample size per state per year (SSY).

### Cancer incidence and mortality data

We obtained the state-wise cancer incidence and mortality data from the publication on the state-wise burden of cancer (India State-Level Disease Burden Initiative Cancer Collaborators, the year 2016) [11]. The state-wise crude incidence rate and mortality rate for cancers (for both genders) were then multiplied by the state population to obtain the crude number of incident cases and deaths. The study also provided the 95% confidence limits for these rates, which were similarly multiplied by the state population to obtain the 95% confidence limits of the number of incident cases and deaths.

The National Cancer Registry Programme (NCRP) has published cancer incidence data for five common cancer sites [12]. We abstracted data for four major cancers, viz. breast cancer, head neck cancers, lung cancer and cervical cancer from the NCRP report [12]. In the NCRP report, statewide population-based incidence data are available for 18 Indian states. For certain states such as Maharashtra and Kerala, more than one PBCR are available. In such a situation, we took the simple average of the reported rates. For the other states and union territories without a PBCR, we used the average crude incidence rate of the 18 states. All incidence rates were taken for the year 2016.

### Geographical access to clinical trial

The key metric for geographical access to clinical trial data was the SSY per 100,000 population when the intervention being studied was preventive (for example, screening trials). This is because these trials are conducted on healthy volunteers. On the other hand, when intervention being studied was non-preventive (e.g. treatment or diagnostic), then the metric was SSY per 1,000 incident cancers. SSY per cancer site (per 1,000 incident cases) for therapeutic intervention studies was also calculated using the same methodology. We adopted this metric as district-wise data on cancer incidence is not available for India. Dividing the SSY by ten allows us to get an estimate of the percentage of newly diagnosed (incident) cancer patients with an available slot in a cancer clinical trial per state.

### Data on health indicators

In order to evaluate the impact of health indicators on the disparity in access to clinical trials, we collected data on the following indicators state-wise:

1. The infant mortality rate from the Open Government Data Platform (OGD) of India (https://data.gov.in/)
2. Literacy rate of the year 2011 from the OGD platform (https://data.gov.in/)
3. Maternal mortality rate (2014–16) from the OGD platform (https://data.gov.in/)
4. The number of cancer centres per 100,000 population. This was obtained by amalgamating data from the list of hospitals which are part of the National Cancer Grid website (https://tmc.gov.in/ncg/index.php/list-of-centers) and the list of licensed radiotherapy centres from the Atomic Energy Regulatory Board of India website (https://www.aerb.gov.in/images/PDF/Radiotheraphy/RSD3.pdf)
5. The number of teaching hospitals per 100,000 population from the National Medical Council website (https://www.nmc.org.in/information-desk/for-students-to-study-in-india/list-of-college-teaching-mbbs ). Presented as
6. Per capita state-wise net domestic product (2017–18) from the OGD platform (https://data.gov.in/)

### Statistical analysis

Statistical analysis was conducted using R. Descriptive statistics included frequencies for categorical variables and median and range for continuous variables. The Chi-square test was used for comparing categorical variables, while the Kruskal–Wallis test was used for continuous variables. Geospatial visualisation of SSY was done using the tmap package [13]. Scatter plots were generated comparing the SSY against the six pre-specified indicators. Locally weighted scatterplot smoothing lines were superimposed to visualise trends. However, given that the total number of states was small (*n* = 36), formal linear regression was not attempted.

## Results

**Figure 1.**
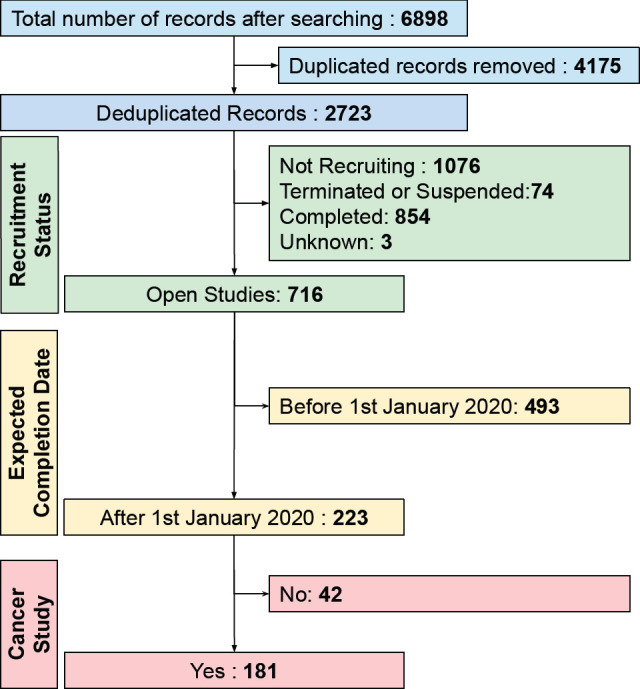
The filtering steps employed for retrieved CTRI records. The expected completion date was calculated by adding the planned trial duration to the date of start of enrolment. Cancer studies were studies related to cancer, and data were recorded for all open studies.

We identified 181 open cancer clinical trials (Figure 1). Table 1 shows the descriptive analysis of these trial records. These trials were registered in CTRI between 2012 and 2020. Full details of 181 trials are available in Appendix 1 in the Supplementary Material.

Amongst the non-interventional studies, there were 6 (12.2%) BA/BE studies and two PMS studies (4.1%), while the rest were observational studies (*n* = 41, 83.7%). Amongst the 96 randomised trials, there was a single cluster-randomised trial (1.0%) and six (6.3%) trials with a cross-over design. Twenty-one trials (21.9%) were placebo-controlled randomised parallel-group trials. Among the interventional studies (*n* = 132), 121 studies were testing a form of treatment. Of these 121, 69 studies (57.0%) were being conducted in the patients being treated with curative intent. Table 2 shows the distribution of the studies as per the treatment setting. Radiotherapy and surgery-related trials together comprised only 39.1% of the all interventional studies being conducted in curative settings (Table 2).

Of the 72 interventional studies investigating systemic treatments, 46 (63.9%) were being conducted in the palliative setting. The most common source of funding was intramural (*n* = 70, 38.7%), followed by industry-sponsored (*n* = 64, 35.4%). Twenty studies (11.04%) had governmental funding, and 27 (14.9%) had other sources of funding.

The median number of studies being conducted in each state was 16.0 (range: 1.0–119.0), and the median total sample size was 7,701 (range: 53–129,878) (Appendix 2 in the Supplementary Material).

**Figure 2.**
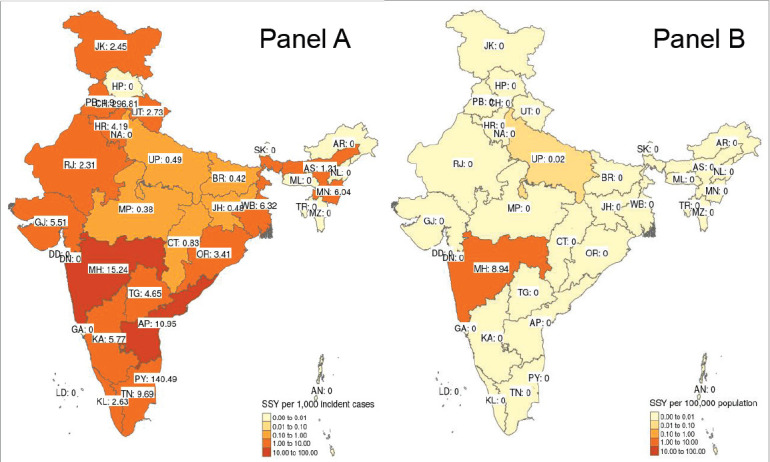
Heat map of inter-state variation in the sample size per year for therapeutic trials (Panel A) and preventive trials (Panel B). Note that for therapeutic trials, the SSY per 1,000 incident cancer cases was calculated while for preventive studies, it was calculated per 100,000 population. Note that each shade of colour scale represents a tenfold change in SSY.

Available SSY per state ranged between 0.00 and 296.81 per 1,000 incident cancer cases (median of 1.02 per 1,000 incidence cancer cases) for therapeutic interventional trials (F) (Figure 2). No studies were available in 13 (13, 35.1%) states or union territories. For interventional studies which were testing a preventive approach, the median SSY was 0.00 per 100,000 population (range: 0.00–8.94 per 100,000). Such studies were ongoing in only two states (5.41%).

**Figure 3.**
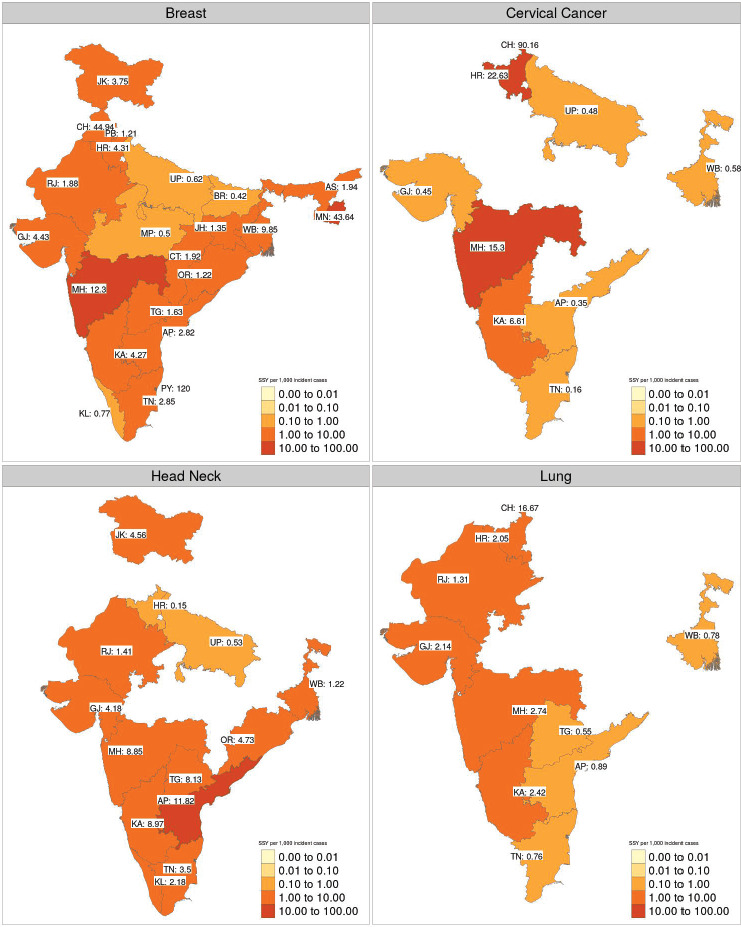
Heatmap of inter-state variation in the sample size per year for therapeutic trials for four major cancer sites. The SSY per 1,000 incident cancer cases is shown in the text. Blank states have no clinical trial running for the specific disease site. Note that each shade of colour scale represents a tenfold change in SSY.

The geographical disparity in access to interventional studies for four major cancer sites is shown in Figure 3. The median SSY per 1,000 incident cases available in interventional therapeutic trials for the four common cancers was as follows (Figure 3):

1. Breast cancer: 2.38 (range: 0.42–120.00 per 1,000 incident cases).
2. Cervical cancer: 0.58 (range: 0.16–90.16 per 1,000 incident cases).
3. Head and Neck cancer: 4.18 (range: 0.15–11.80 per 1,000 incident cases).
4. Lung cancer: 1.68 (range: 0.55–16.67 per 1,000 incident cases).

Even for the commonest cancer in Indian females (Breast Cancer), the highest SSY is 120.00 per 1,000 (Puducherry) implying that only 12% of the incident breast cancer patients will have a clinical trial slot available in that state.

**Figure 4.**
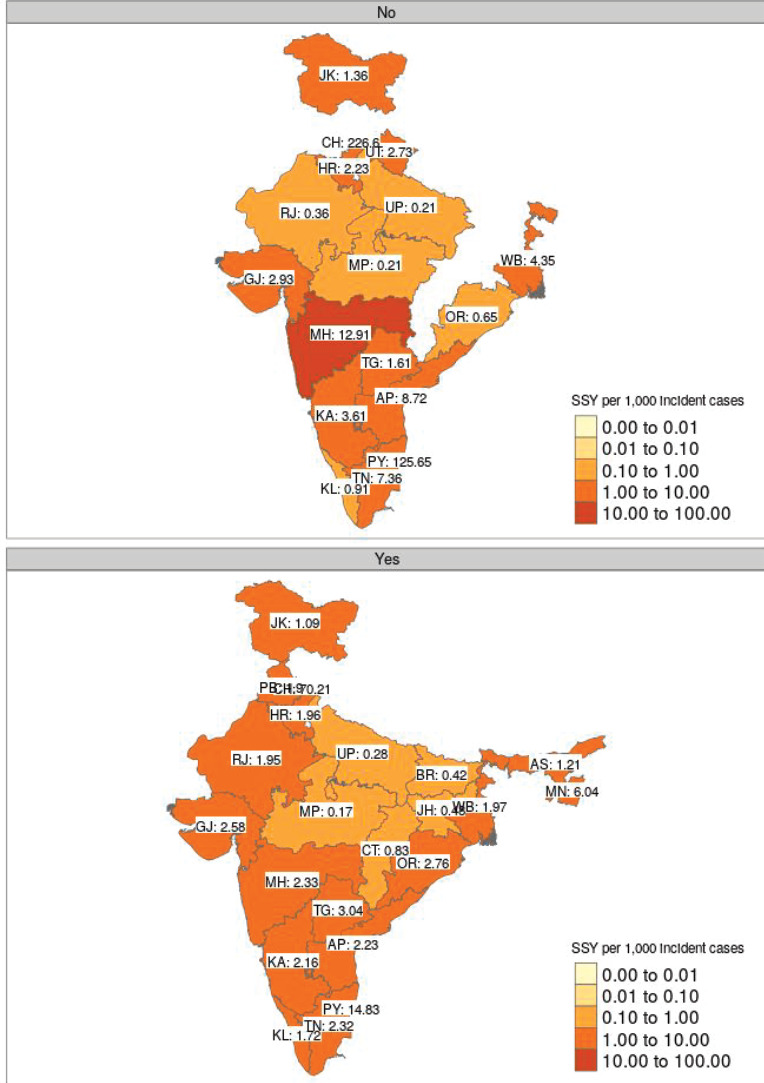
Faceted heatmap of variation in SSY as per the sponsor type (academic on the top versus industry-sponsored in the bottom). The SSY per 1,000 incident cancer cases is shown in the text. Blank states have no clinical trial running for the specific disease site. Note that each shade of colour scale represents a tenfold change in SSY.

Figure 4 shows the geographical disparity in access to interventional studies based on the type of trial sponsorship. The median SSY in academic studies was 2.73 (range: 0.21–226.60), while it was 1.97 (range: 0.17–70.21) for industry-sponsored research studies. However, as can be seen from the heatmap, geographical access to studies is likely to be more heterogeneous for academic studies.

**Figure 5.**
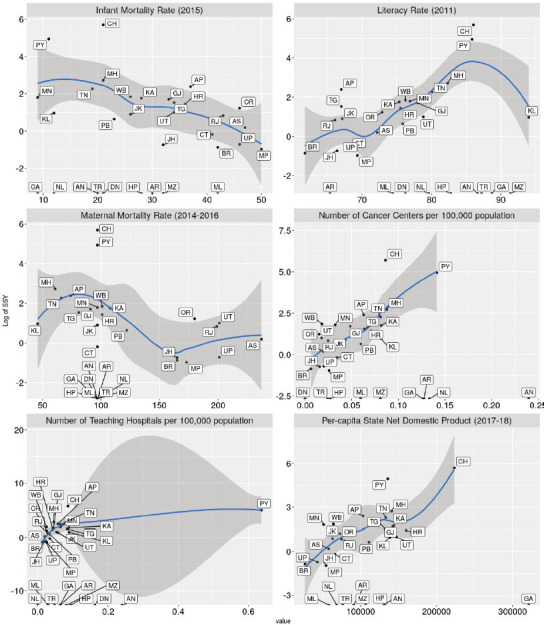
Faceted scatterplots of the relationship between SSY for therapeutic trials and key health indicators (infant mortality rate 2015, literacy rate 2011, maternal mortality rate (2014–16), number of cancer centres per 100,000 population in the state, number of teaching hospitals per 100,000 population and per-capita state net domestic product). Labels indicate state abbreviation. Loess smoothing line with 95% confidence intervals superimposed on the scatter plot. Note that SSY is shown on the Y-axis in the log scale. Note that several states with 0 SSY are depicted at the bottom.

**Figure 6.**
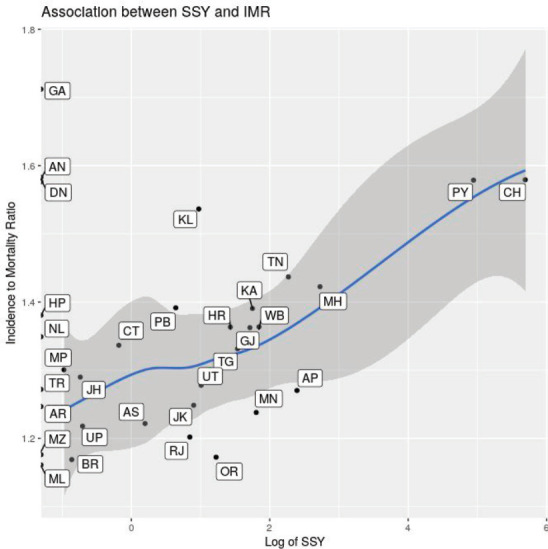
Scatter plot showing the association between state-wise incidence to mortality ratio and the log of SSY for therapeutic interventional studies. Labels indicate state abbreviation. Loess smoothing line with 95% confidence intervals superimposed on the scatter plot. Note that SSY is shown on the X-axis in the log scale.

Scatterplots of the relationship between the SSY and the indicators are shown in Figure 5. There seems to be an association with the state-wise per-capita net domestic product and the number of cancer centres per 100,000 population, but in all plots outliers are present. The association between cancer incidence to mortality ratio and the SSY for therapeutic interventional studies is shown in Figure 6. States with a higher incidence to mortality ratio tend to have a higher SSY per 1,000.****

## Discussion

In the current analysis, we found that there is a high disparity or difference or variation across geographic locations in India in accessing clinical trials by the cancer patients, particularly in four common malignancies, including breast cancer, cervical cancer, head and neck cancer and lung cancer.

There is nearly a thousandfold difference in the SSY between the states with the smallest and the highest SSY in therapeutic interventional studies. States in the North-eastern part of the country have fewer available clinical trial slots for patients. Tobacco associated cancers account for about one-third of all cancers in India [8]. It is, therefore, surprising to see that active intervention studies investigating a preventive approach were being conducted in only two states in the country. Across the nation, however, access to cancer clinical trials remains poor. Even in the state with the highest SSY of 55.21 per 1,000 incident cases (Delhi), by definition, a clinical trial slot is available for only 5.5% of the new cancer patients diagnosed every year in the state. Note there are two union territories with a higher SSY—Chandigarh with SSY of 296 per 1,000 incident cases and Puducherry with SSY of 141 per 1,000 incident cases.

The disparity is even starker when cancer sites are considered. For example, therapeutic interventional studies are available for brain tumour patients in only two states in the country. The true magnitude of disparity is likely to be magnified by the fact that most of the cancer centres in which these studies are available are located in urban areas of the country, in addition to the other barriers to research [14–16].

The reasons behind the disparity are possibly multi-fold and out of the scope of the present analysis. As SSY is a state-level metric, the total number of values available is limited to permit an adequate regression model. Unfortunately, district-level data of cancer incidence is not available in India, which would have permitted evaluation of the influence of the selected indicators on the disparity in access. One factor that shows a relationship is the per-capita state net domestic product, where the higher per-capita net domestic product seems to be associated with a higher SSY. State literacy rate also seems to have a monotonous relationship. However, given the small number of states and the presence of outliers, a definite relationship cannot be established.

**Figure 7.**
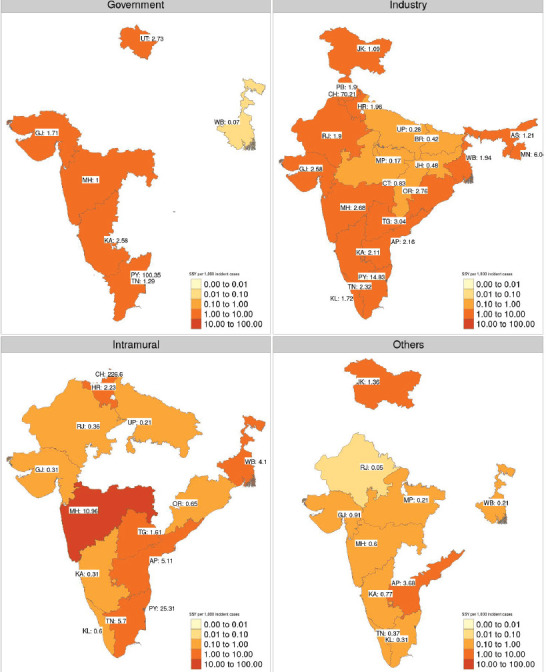
Heatmap of SSY for interventional therapeutic studies faceted by the source of funding. The SSY per 1,000 incident cancer cases is shown in the text. Blank states have no clinical trial funded by the specific source. Note that each shade of colour scale represents a tenfold change in SS.

Further exploration of funding sources also reveals that disparity persists across funding sources also (Figure 7). Government-funded therapeutic interventional studies were available in only six states (6, 16%). Interestingly intra-mural funded studies seem to be spread out more uniformly across the country. Industry-sponsored studies seem to have the least geographical disparity. This highlights the importance of industry-sponsored studies in our country where healthcare research spending is quite limited. For example, the annual budgetary allocation for the Department of Health Research which is broadly responsible for conducting health research for 2020–21 is Rs. 2,100 crore (approximately 283 million USD). This corresponds to a per-capita figure of 0.22 USD (assuming a population of 1.3 billion) [17].

The association between the incidence to mortality ratio and the SSY is intriguing, but a causative role cannot be attributed. Given the overall low number of cancer clinical trial seats, it is unlikely that participation in clinical trials itself is responsible for the improved outcomes in the states with higher SSY. Better healthcare infrastructure and facilities in these states that facilitate the conduct of cancer clinical trials may be responsible for such an association with improved incidence mortality ratio.

Inequities in geographical access to cancer care have been demonstrated to be associated with increasing stage at diagnosis, poor compliance with treatment, worse outcomes and quality of life [18]. Syed *et al* [19] have additionally shown that such disparities disproportionately affect minorities and those with lower incomes. Additionally, this is a barrier to fair representation in clinical trials. In a statewide survey of oncology patients in Pennsylvania, only 37% of patients indicated that they would be willing to travel in order to participate in a clinical trial [20]. Similar findings were demonstrated by Lara *et al* [21] in a prospective study of patients at the University of California Davis Cancer Center, where the distance from the cancer centre was the second most common reason cited for not participating in a clinical trial. Whether this is true for Indian patients needs to be investigated further.

A recent paper demonstrated that unequal geographic access to clinical trials exists even in the USA [median clinical trial seats per 1,000 residents: 0.64, interquartile range (IQR): 0.25–1.01] [22]. In an analysis by Galsky *et al* [23], it was found that 45.6%, 50.2%, 52.2% and 38.4% of the patients with metastatic breast, prostate, colorectal and non-small cell lung cancers, respectively, will have to drive more than 1 hour to access a clinical trial site. It should be noted that Indian cancer patients routinely travel much longer distances to avail quality cancer care [24, 25]. Carneiro *et al* [26] have shown that the number of interventional cancer clinical trials per 100,000 population in the country ranges between 0.14 and 10.7 in Europe. In India, where we identified only 132 open interventional clinical trials, the number of open interventional trial entries per 100,000 population is only 0.01 (for a population of 1.3 billion).

The strengths of this study are a detailed evaluation of the intra-state geographic disparity in access to clinical trials using publicly available high-quality data. To the author’s knowledge, this is the first analysis of this kind. Disparities exist across study types and are magnified when specific cancer types are evaluated.

It is only natural that interventional cancer clinical trials are conducted in cancer centres—and therefore states and small union territories with fewer cancer centres have fewer clinical trials available. However, just the presence of cancer centres is not enough. Availability of research funding, personnel and infrastructure to conduct trials is likely to be non-uniformly distributed too [27]. In less endowed states, the priorities of cancer centres lean towards provision of cancer care rather than research. Academic incentives for research may also be non-uniformly distributed. Healthcare literacy in the population may be influenced by the literacy rates and income. States with a high literacy rate do not automatically have a higher number of clinical trials available.

The socio-economic disparity and cultural diversity of our nation are likely to influence the conduct and access to preventive clinical trials [28]. Agarwal *et al* [29] report several barriers to conducting such studies like loss to follow up due to the migratory nature of the workforce, socioeconomic and cultural issues (gender disparity, casteism and stigma of disease) and lack of access to primary health care services. Similar findings have been reported by Joseph *et al* [30]. Securing long-term funding from the government is also challenging given the lack of social and political demand for health care in the country [31].

Our study has several limitations. First, our analysis is limited to studies registered in the CTRI. We chose to restrict the search to the CTRI database as the Central Drugs Standard Control Organisation (CDSCO) has mandated the registration of all clinical trials in CTRI since 15 June 2009 [32, 33]. Second, geographic access has been estimated based on state, while clinical trials are conducted in specific institutes. Unfortunately, institute wise cancer statistics are not available in the public domain for all cancer institutes across the country. However, the geographic disparity will likely be more magnified if an institute level analysis is conducted as the majority of cancer centres in India are located in urban areas. Similarly, as we have considered only incident cases in the denominator, which is likely to be highly conservative as the prevalence of several cancers is likely to be higher than the incidence. Third, we have used the incidence data from two sources which rely on PBCR and hospital-based cancer registries for estimates of new cancer case burden. However, registry coverage across the nation is not homogenous. Fourthly, key information about disease characteristics like stage information is inconsistently recorded in the trial registration data which limits our ability to evaluate such factors in granular details. Finally, we restricted our analysis to open trials where studies whose planned duration was such that they should have closed by 1 January 2020 were considered as completed. It is likely that some of these trials may still be open to recruitment.****

## Conclusion

Significant geographical variability in access to cancer clinical trials exists in India. Fewer than 10% of newly diagnosed cancer patients will have access to a therapeutic cancer clinical trial in the same state. Further studies are needed on methods to reduce and mitigate such disparities.

## Funding statement

No intramural or extramural funding was available for this study.

## Figures and Tables

**Table 1.** Trial characteristics for interventional and non-interventional studies.

	Interventional study	Non-interventional study	Total (*N* = 181)	*p*-value
**Total number of studies**	132	49	181	
**Total sample size**				0.021
Median	71	120	88	
Range	5.0–110000.0	1.0–30000.0	1.0–110000.0	
**Total state-wise sample size**				0.007
Median	40	100	50	
Range	2.0–110000.0	1.0–30000.0	1.0–110000.0	
**State-wise sample size per year (SSY)**				<0.001
Median	13	43	18	
Range	0.0–11008.0	0.0–15010.0	0.0–15010.0	
**Randomised trial**				<0.001
No	42 (31.8%)	43 (87.8%)	85 (47.0%)	
Yes	90 (68.2%)	6 (12.2%)	96 (53.0%)	
**Nations**				0.016
Indian	86 (65.2%)	41 (83.7%)	127 (70.2%)	
Multinational	46 (34.8%)	8 (16.3%)	54 (29.8%)	
**Industry-sponsored**				0.13
No	81 (61.4%)	36 (73.5%)	117 (64.6%)	
Yes	51 (38.6%)	13 (26.5%)	64 (35.4%)	
Post-graduate thesis				0.002
No	105 (79.5%)	28 (57.1%)	133 (73.5%)	
Yes	27 (20.5%)	21 (42.9%)	48 (26.5%)	
**Trial duration**				0.001
Median	3.5	2	3	
Range	1.0–15.0	0.0–10.0	0.0–15.0	
**Multi-centric trial**				0.017
No	71 (53.8%)	36 (73.5%)	107 (59.1%)	
Yes	61 (46.2%)	13 (26.5%)	74 (40.9%)	
**Sites enrolling**				0.017
Median	1	1	1	
Range	1.0–52.0	1.0–17.0	1.0–52.0	
**States enrolling**				0.015
Median	1	1	1	
Range	1.0 - 21.0	1.0 - 9.0	1.0 - 21.0	
**States enrolling category**				0.077
>5	34 (27.0%)	8 (16.3%)	42 (24.0%)	
1	75 (59.5%)	38 (77.6%)	113 (64.6%)	
1–5	17 (13.5%)	3 (6.1%)	20 (11.4%)	
**Cancer stage**				0.196
Metastatic	36 (27.3%)	9 (18.4%)	45 (24.9%)	
Non-metastatic	64 (48.5%)	22 (44.9%)	86 (47.5%)	
All stages	32 (24.2%)	18 (36.7%)	50 (27.6%)	
**Cancer site**				0.126
Brain	9 (6.8%)	2 (4.1%)	11 (6.1%)	
Breast	22 (16.7%)	5 (10.2%)	27 (14.9%)	
Gastrointestinal	17 (12.9%)	12 (24.5%)	29 (16.0%)	
Genitourinary	6 (4.5%)	6 (12.2%)	12 (6.6%)	
Gynaecological	16 (12.1%)	2 (4.1%)	18 (9.9%)	
Head Neck	21 (15.9%)	5 (10.2%)	26 (14.4%)	
Haematological	14 (10.6%)	4 (8.2%)	18 (9.9%)	
Lung	12 (9.1%)	4 (8.2%)	16 (8.8%)	
Multiple types	13 (9.8%)	9 (18.4%)	22 (12.2%)	
Sarcoma	2 (1.5%)	0 (0.0%)	2 (1.1%)	

Individual study descriptions are available in Appendix 1. Non-interventional studies include study types like observational studies (*n* = 42), BA/BE studies (*n* = 6) and PMS studies (*n* = 2). *p*-values calculated using Chi-square test for categorical variables and Kruskal–Wallis test for continuous variables. Note that states with no ongoing studies have been excluded from the analysis.

**Table 2.** Table showing the type of treatments being investigated in interventional studies.

	Curative (*n* = 69)	Palliative (*n* = 50)	Both (*n* = 2)	Total (*n* = 121)	*p*-value
Radiotherapy	17 (24.6%)	2 (4.0%)	0 (0.0%)	19 (15.7%)	<0.001
Supportive care	16 (23.2%)	2 (4.0%)	2 (100.0%)	20 (16.5%)	
Surgery	10 (14.5%)	0 (0.0%)	0 (0.0%)	10 (8.3%)	
Systemic therapy	26 (37.7%)	46 (92.0%)	0 (0.0%)	72 (59.5%)	

Supportive care includes interventions related to pain and palliative care. Both = Patients with both curative and palliative intent included in the study. *p*-value calculated using the Chi-square test.

## Appendix 1 trial list

The trial list is available at https://doi.org/10.6084/m9.figshare.13366238.v1.

## Appendix 2 state-wise sample size

**Table:**

State	Number of Incident Cases	Total Number of Studies	Total Sample Size	SSY Per 1000	95% Lower CI of Number of Incident Cases	Upper 95% CI for SSY per 1000	95% Upper CI of Number of Incident Cases	Lower 95% CI for SSY per 1000
CHANDIGARH	940	13.00	857.71	296.81	902.00	309.31	982.00	284.11
PUDUCHERRY	1146	5.00	292.45	140.49	1101.00	146.23	1199.00	134.28
DELHI	19254	51.00	2364.54	55.21	16185.00	65.68	20788.00	51.14
MAHARASHTRA	98762	77.00	7526.88	15.24	95437.00	15.77	104180.00	14.45
ANDHRA PRADESH	41290	25.00	1113.12	10.95	39996.00	11.30	44147.00	10.24
TAMIL NADU	64530	40.00	2482.96	9.69	61728.00	10.13	69668.00	8.97
WEST BENGAL	85066	42.00	3044.59	6.32	82178.00	6.55	87756.00	6.13
MANIPUR	1988	1.00	12.63	6.04	1907.00	6.29	2223.00	5.40
KARNATAKA	68644	39.00	1216.08	5.77	66076.00	5.99	71752.00	5.52
GUJARAT	48415	30.00	827.72	5.51	46563.00	5.73	51226.00	5.21
TELANGANA	28577	17.00	210.51	4.65	27318.00	4.87	30427.00	4.37
HARYANA	29135	18.00	318.10	4.19	27584.00	4.42	31307.00	3.90
ODISHA	38754	14.00	210.99	3.41	37270.00	3.54	42045.00	3.14
UTTARAKHAND	10238	1.00	42.00	2.73	9676.00	2.89	10823.00	2.59
KERALA	48301	12.00	332.34	2.63	38984.00	3.26	50550.00	2.51
JAMMU AND KASHMIR	11005	2.00	65.13	2.45	10616.00	2.54	11658.00	2.32
RAJASTHAN	58830	21.00	320.33	2.31	56642.00	2.40	61342.00	2.22
PUNJAB	25771	6.00	61.77	1.90	24083.00	2.03	26705.00	1.83
ASSAM	32118	3.00	40.54	1.21	30907.00	1.26	33613.00	1.16
CHHATTISGARH	24138	3.00	22.92	0.83	23313.00	0.86	25345.00	0.79
UTTAR PRADESH	187927	13.00	144.75	0.49	180553.00	0.52	196491.00	0.47
JHARKHAND	24816	1.00	12.63	0.48	23967.00	0.50	25897.00	0.46
BIHAR	67267	4.00	43.88	0.42	64272.00	0.44	70512.00	0.40
MADHYA PRADESH	70933	2.00	42.63	0.38	68031.00	0.40	73153.00	0.37
HIMACHAL PRADESH	6826	0.00	0.00	0.00	6468.00	0.00	7281.00	0.00
TRIPURA	2877	0.00	0.00	0.00	2769.00	0.00	3144.00	0.00
MEGHALAYA	2741	0.00	0.00	0.00	2636.00	0.00	2892.00	0.00
NAGALAND	1582	0.00	0.00	0.00	1523.00	0.00	1692.00	0.00
GOA	1539	0.00	0.00	0.00	1467.00	0.00	1616.00	0.00
MIZORAM	1508	0.00	0.00	0.00	1312.00	0.00	1559.00	0.00
ARUNACHAL PRADESH	1233	0.00	0.00	0.00	1121.00	0.00	1277.00	0.00
DADRA AND NAGAR HAVELI	499	0.00	0.00	0.00	480.00	0.00	522.00	0.00
ANDAMAN AND NICOBAR ISLANDS	338	0.00	0.00	0.00	325.00	0.00	354.00	0.00

## References

[1] Tejeda HA, Green SB, Trimble EL (2002). Representation of African-Americans, hispanics, and whites in National Cancer Institute cancer treatment trials. J Natl Cancer Inst.

[2] Sateren WB, Trimble EL, Abrams J (2002). How sociodemographics, presence of oncology specialists, and hospital cancer programs affect accrual to cancer treatment trials. J Clin Oncol.

[3] Murthy VH, Krumholz HM, Gross CP (2004). Participation in cancer clinical trials: race-, sex-, and age-based disparities. JAMA.

[4] Wong AR, Sun V, George K (2020). Barriers to participation in therapeutic clinical trials as perceived by community oncologists. JCO Oncol Pract.

[5] Unger JM, Vaidya R, Hershman DL (2019). Systematic review and meta-analysis of the magnitude of structural, clinical, and physician and patient barriers to cancer clinical trial participation. J Natl Cancer Inst.

[6] Mills EJ, Seely D, Rachlis B (2006). Barriers to participation in clinical trials of cancer: a meta-analysis and systematic review of patient-reported factors. Lancet Oncol.

[7] Ramaswami R, Paulino E, Barrichello A (2018). Disparities in breast, lung, and cervical cancer trials worldwide. J Glob Oncol.

[8] Mathur P, Sathishkumar K, Chaturvedi M (2020). Cancer statistics, 2020: report from national cancer registry programme, India. JCO Glob Oncol.

[9] Ferlay J, Colombet M, Soerjomataram I (2019). Estimating the global cancer incidence and mortality in 2018: GLOBOCAN sources and methods. Int J Cancer.

[10] Roy AM, Mathew A (2019). Audit of cancer clinical trials in India. J Glob Oncol.

[11] India State-Level Disease Burden Initiative Cancer Collaborators (2018). The burden of cancers and their variations across the states of India: the Global Burden of Disease Study 1990–2016. Lancet Oncol.

[12] Indian Council of Medical Research Report of National Cancer Registry Programme 2020 [Internet]. National Centre for Disease Informatics and Research. https://www.ncdirindia.org/All_Reports/Report_2020/default.aspx.

[13] Tennekes M (2018). tmap: thematic maps in R. J Stat Softw.

[14] Burt T, Sharma P, Dhillon S (2014). Clinical research environment in India: challenges and proposed solutions. J Clin Res Bioeth.

[15] Dandekar M, Trivedi R, Irawati N (2016). Barriers in conducting clinical trials in oncology in the developing world: a cross-sectional survey of oncologists. Indian J Cancer.

[16] Alemayehu C, Mitchell G, Nikles J (2018). Barriers for conducting clinical trials in developing countries- a systematic review. Int J Equity Health.

[17] Demand for Grants 2020-21 Analysis: Health and Family Welfare [Internet]. https://www.prsindia.org/parliamenttrack/budgets/demand-grants-2020-21-analysis-health-and-family-welfare.

[18] Ambroggi M, Biasini C, Del Giovane C (2015). Distance as a barrier to cancer diagnosis and treatment: review of the literature. Oncologist.

[19] Syed ST, Gerber BS, Sharp LK (2013). Traveling towards disease: transportation barriers to health care access. J Community Health.

[20] Meropol NJ, Buzaglo JS, Millard J (2007). Barriers to clinical trial participation as perceived by oncologists and patients. J Natl Compr Canc Netw.

[21] Lara PN, Higdon R, Lim N (2001). Prospective evaluation of cancer clinical trial accrual patterns: identifying potential barriers to enrollment. J Clin Oncol.

[22] Feyman Y, Provenzano F, David FS (2020). Disparities in clinical trial access across US urban areas. JAMA Netw Open.

[23] Galsky MD, Stensland KD, McBride RB (2015). Geographic accessibility to clinical trials for advanced cancer in the United States. JAMA Intern Med.

[24] Karim S, Del Paggio JC, Berry SR (2016). Cancer care in South India: perspectives from visiting Canadian oncologists. Curr Oncol.

[25] Ballari N, Miriyala R, Jindia T (2018). Time, distance and economics influencing cancer care: experience from a regional cancer center in India. JGO.

[26] Carneiro A, Amaral TMS, Brandao M (2020). LBA66_PR Disparities in access to oncology clinical trials in Europe in the period 2009–2019. Ann Oncol.

[27] Sullivan R, Badwe RA, Rath GK (2014). Cancer research in India: national priorities, global results. Lancet Oncol.

[28] Thulaseedharan JV, Frie KG, Sankaranarayanan R (2019). Challenges of health promotion and education strategies to prevent cervical cancer in India: a systematic review. J Educ Health Promot.

[29] Agrawal T, Fathima FN, Hegde SKB (2015). Challenges in conducting community-based trials of primary prevention of cardiovascular diseases in resource-constrained rural settings. WHO South East Asia J Public Health.

[30] Joseph LM, Lekha TR, Boban D (2019). Perceived facilitators and barriers of enrolment, participation and adherence to a family based structured lifestyle modification interventions in Kerala, India: a qualitative study. Wellcome Open Res.

[31] Kumar R (2015). Lack of social or political demand for good health care in India: impact on unfolding universal health coverage. J Family Med Prim Care.

[32] Sil A, Das NK (2013). How to register a clinical trial in India?. Indian J Dermatol.

[33] Ministry of Health and Family Welfare (2019). The Gazette of India: extraordinary authority.

